# Application of Ultrasound in a Closed System: Optimum Condition for Antioxidants Extraction of Blackberry (*Rubus fructicosus*) Residues

**DOI:** 10.3390/molecules21070950

**Published:** 2016-07-21

**Authors:** Quinatzin Y. Zafra-Rojas, Nelly S. Cruz-Cansino, Aurora Quintero-Lira, Carlos A. Gómez-Aldapa, Ernesto Alanís-García, Alicia Cervantes-Elizarrarás, Norma Güemes-Vera, Esther Ramírez-Moreno

**Affiliations:** 1 Instituto de Ciencias Agropecuarias, Universidad Autónoma del Estado de Hidalgo, Av. Universidad Km 1, Rancho Universitario, Tulancingo de Bravo, Hidalgo 43600, Mexico; zafhry@hotmail.com (Q.Y.Z.-R.); auroraql@yahoo.com.mx (A.Q.-L.); elizarraras.alice@yahoo.com.mx (A.C.-E.); njgv2002@yahoo.com.mx (N.G.-V.); 2 Centro de Investigación Interdisciplinario, Área Académica de Nutrición, Instituto de Ciencias de la Salud, Universidad Autónoma del Estado de Hidalgo, Circuito Actopan-Tilcuautla s/n. Ex hacienda La Concepción, San Agustín Tlaxiaca, Hidalgo 42160, Mexico; ncruz@uaeh.edu.mx (N.S.C.-C.); ealanisg70@yahoo.com.mx (E.A.-G.); 3 Centro de Investigaciones Químicas, Instituto de Ciencias Básicas e Ingeniería, Universidad Autónoma del Estado de Hidalgo, Centro Universitario, Carretera Pachuca-Tulancingo Km 4.5, Mineral de la Reforma, Hidalgo 42183, Mexico; cgomeza@uaeh.edu.mx

**Keywords:** blackberry, residues, ultrasound, extraction, antioxidants

## Abstract

Blackberry processing generates up to 20% of residues composed mainly of peel, seeds and pulp that are abundant in flavonoids. The objective of this study was to optimize the ultrasound conditions, in a closed system, for antioxidants extraction, using the response surface methodology. Blackberry (*Rubus fructicosus*) residues were analyzed for total phenolics, total anthocyanins, and antioxidant activity by ABTS and DPPH. The selected independent variables were ultrasound amplitude (X_1_: 80%–90%) and extraction time (X_2_: 10–15 min), and results were compared with conventional extraction methods. The optimal conditions for antioxidants extraction were 91% amplitude for 15 min. The results for total phenolic content and anthocyanins and antioxidant activity by ABTS and DPPH were of 1201.23 mg gallic acid equivalent (GAE)/100 g dry weight basis (dw); 379.12 mg/100 g·dw; 6318.98 µmol Trolox equivalent (TE)/100 g·dw and 9617.22 µmol TE/100 g·dw, respectively. Compared to solvent extraction methods (water and ethanol), ultrasound achieved higher extraction of all compounds except for anthocyanins. The results obtained demonstrated that ultrasound is an alternative to improve extraction yield of antioxidants from fruit residues such as blackberry.

## 1. Introduction

The blackberry fruit belongs to the *Rosaceae* family and the genus *Rubus*. It has a complex genetic background, a diverse number of species and different growth characteristics [1]. Blackberry is widely produced in North and South America, Europe, Asia, Oceania and Africa [2]. Fruit weight varies from 3 to 12 g depending on the variety, and it is comprised of several drupelets, each containing one seed [3]. Blackberries are of great interest due to their high content of polyphenols including anthocyanins that contribute to their high antioxidant activity [4]. Evidence suggests that berry phytochemicals can regulate the activity of enzymes, modulate nuclear receptors, gene expression, and subcellular signaling pathways, and repair DNA oxidative damage [5,6]. These functional characteristics may benefit human health through their antioxidant, anticancer and anti-inflammatory properties [7,8,9].

This fruit is mostly consumed fresh due to being highly perishable; therefore, it is either frozen or thermally processed, depending on its use, and sold individually quick-frozen, bulk-frozen, pureed, freeze-dried, and as juice or concentrate [3]. The food industry use blackberries for the production of supplements, ice cream, jam, marmalade and other confectioneries [10]. This industrialization can generate up to 20% of residues composed mainly of peel, seeds and pulp [11], which are an abundant source of flavonoids, colorants and pectin [12].

The extraction of added-value components from fruit and vegetable residues is an alternative to use materials that are typically discarded. The quality of the extracts depends largely on the employed extraction technique; the most common are solvent extraction methods that use acidified methanol or ethanol [13]. Methanol is the most efficient but is also toxic, so ethanol is preferred by the food industry [14]. Emerging technologies such as ultrasound have been successfully used as alternative extraction methods of bioactive compounds from plant matrices.

Ultrasound (US) is a sound wave transmitted at a frequency higher than the audible one of 20 kHz [15]. The food industry applies low (intensities <1 W/cm^2^ and frequencies >100 kHz) and high (intensities >1 W/cm^2^ and frequency of 20–100 kHz) energy ultrasound. The first one is used as a nondestructive analytical technique to monitor the composition and physicochemical properties of food during processing and storage, while the high intensity ultrasound is used to impact the physical, chemical and biological properties of foods during processing and preservation [16]. Two different ultrasonic techniques can be applied, one in which the sample is submerged in an ultrasonic water bath [17], and the other which uses a sonicator probe directly on the liquid sample such as fruit juice [18,19]. Cavitation is one phenomenon occurring during ultrasound treatment in the form of micro-bubbles generated by pressure changes, these micro-bubbles collapse violently [20], facilitating the release of several compounds including antioxidants [19]. The objective of this study was to determine the optimum ultrasound conditions in a closed system for antioxidants extraction (total phenolic content and anthocyanins) and antioxidant activity from blackberry (*Rubus fructicosus*) residues using response surface methodology and to compare this technology with conventional extraction methods.

## 2. Results and Discussion

### 2.1. Modeling of the Extraction Process

In order to optimize the extraction process based on the total phenolic content (TPC), anthocyanins and antioxidant activity from blackberry residues, a central composite rotatable design was used. Table 1 shows the effect of the ultrasound (US) amplitude level and extraction time on TPC, anthocyanins, ABTS and DPPH values which had intervals of 933–1438 mg GAE/100 g·dw, 332–378 mg/100 g·dw, 3478–6953 μmol TE/100 g·dw and 7050–9571 μmol TE/100 g·dw, respectively.

The regression coefficients of the model for each response and the results of the analysis of variance (ANOVA) are summarized in Table 2. The high coefficients of multiple determination (*R*^2^ ≥ 0.94) for TPC, anthocyanins, ABTS and DPPH suggest that the applied model was adequate for the experimental results at the 95% confidence level. The adjusted-*R*^2^ is a regression coefficient that adjusts the mathematical model based on the number of coefficients. It is used to compare models with different numbers of fixed variables and to test the suitability to the regression coefficient [21]. The adj-*R*^2^ for each dependent variable was ≥ 0.89, which indicates a high degree of correlation between the observed and the predicted data. Furthermore, the low coefficient of variation (CV < 10%) for each model (Table 2) suggests a good model fit and reproducibility.

### 2.2. Effect of Amplitude and Time on Total Phenolic Content and Anthocyanins

**Figure 1:**
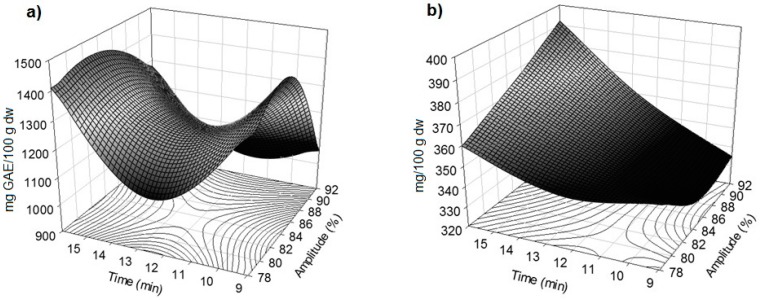
Response surface plots showing the effect of amplitude and time on (**a**) total phenolic content (mg gallic acid equivalent/100 g dry weight basis) and (**b**) anthocyanins (mg cyanidin-3-glucoside/100 g dry weight basis) of blackberry residues extracted by ultrasound.

The linear amplitude term (β_1_) for the extraction of TPC was significant at *p* < 0.01, while the quadratic terms for amplitude and time (β_11_, β_22_, respectively) were highly significant (*p* < 0.0001). Amplitude terms, linear (β_1_) and quadratic (β_11_), had negative coefficients whereas the quadratic time term (β_22_) was positive (Table 2). The influence of US amplitude and extraction time on TPC is shown in Figure 1a.

The TPC decreased with the increase of the applied amplitude while the opposite effect was observed with the extraction time, so that a higher release of phenolic content was achieved at the maximum treatment time. This effect of time may be attributed to the extended contact time between the solvent and the residues that improved the diffusion of phenolic compounds [22]. Similar results were found in red grape skins after exposure time to the solvent [23].

The linear term of amplitude (β_1_) and the interaction between amplitude and time (β_12_) for anthocyanins were significant at *p* < 0.01 (Table 2), while the linear term of time (β_2_) was highly significant (*p* < 0.0001). The maximum anthocyanins extraction was achieved when the extraction time increased (Figure 1b). Similarly, a higher extraction of anthocyanins from wine grape skins was also achieved with extended sonication [24].

### 2.3. Effect of Amplitude and Time on the Antioxidant Activity

**Figure 2:**
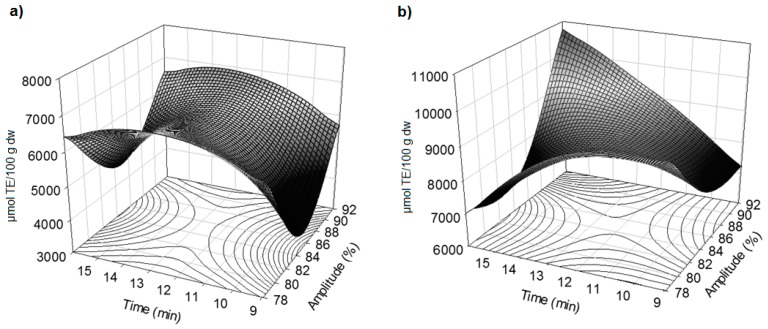
Response surface plots showing the effect of amplitude and time on (**a**) antioxidant activity by the ABTS method (µmol of Trolox equivalent/100 g dry weight basis) and (**b**) DPPH method (µmol of Trolox equivalent/100 g dry weight basis) obtained by ultrasound from blackberry residues.

The antioxidant activity of blackberry residues was determined by the ABTS and DPPH methods. For ABTS, the linear term of time (β_2_) and the quadratic term of amplitude (β_11_) were significant at *p* < 0.05 and *p* < 0.01, respectively, whereas the quadratic term of time (β_22_) was highly significant (*p* < 0.0001) (Table 2). A study with Andean blackberry, in which ultrasound was used as a pretreatment of convection drying, also found that the quadratic term of amplitude and the linear term of time had a significant effect on the antioxidant activity measured by FRAP [25]. Figure 2a shows that the maximum antioxidant activity by ABTS was achieved between 12–13 min of US. The quadratic term of time (β_22_) for ABTS presented a similar significance level as the TPC, maybe because when ABTS^•+^ readily reacts with H-atom donors, such as phenolics, it is converted into a non-colored form of ABTS [26].

The quadratic terms of amplitude (β_11_) and time (β_22_) significantly influenced (*p* < 0.01 and *p* < 0.001, respectively) the free radical scavenging activity measured by DPPH, while the effect of the interaction between amplitude and time (β_12_) was highly significant at *p* < 0.0001. This demonstrates that the increase of amplitude and time allowed the high antioxidant activity by DPPH (Table 2 and Figure 2b). Romero [25] reported a similar effect of the quadratic term of amplitude and its interaction with time over the antioxidant activity measured by FRAP. Our results suggest that the increase of US amplitude caused more damage to the cell walls so that antioxidant compounds, such as phenolics, were released into the solvent [27]. Extraction time also improved the release of these compounds which are responsible for greater trapping of free radicals [22]. In contrast to ABTS, DPPH does not react with flavonoids without the OH-groups in the B-ring, and aromatic acids contains only one OH-group [28,29]. Anthocyanins and antioxidant activity by DPPH had a significant effect in the interaction of the independent variables; this suggests that the high antioxidant activity could be attributed to the anthocyanins.

### 2.4. Optimization of Ultrasound Extraction Conditions and Model Validation

**Figure 3:**
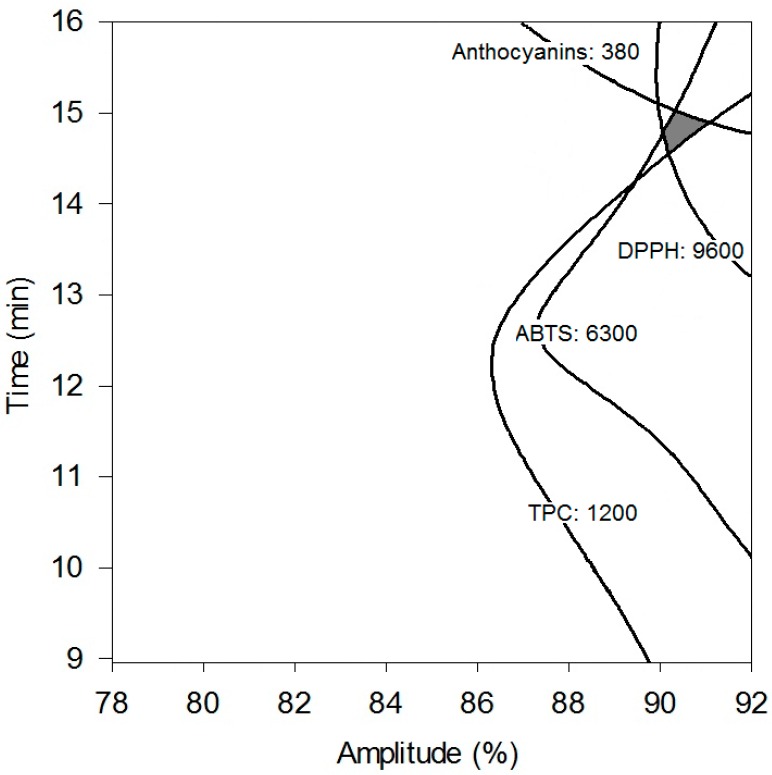
Superimposing contour plots for the response variables (TPC: mg GAE/100 g·dw; Anthocyanins: mg/100 g·dw; ABTS: µmol TE/100 g·dw and DPPH: µmol TE/100 g·dw) as affected by amplitude and time.

The optimization of the extraction process was performed, superimposing the obtained contour plots of each response variable using the surface methodology. The selected responses were TPC, anthocyanins content and antioxidant activity (ABTS, DPPH). The processing conditions corresponding to the highest values of all the dependent variables were used as the criteria to optimize the US process, according to the predicted values obtained by the mathematical model for each response. The area corresponding to the optimal extraction conditions was the interval of the 90%–91% amplitude level and 14–15 min; therefore, the selected conditions were 91% amplitude for 15 min. The obtained values for these conditions were 1200 mg GAE/100 g·dw and 380 mg/100 g·dw for TPC and anthocyanins, respectively, and 6300 and 9600 µmol TE/100 g·dw for antioxidant activity measured by ABTS and DPPH, respectively (Figure 3).

To validate the mathematical model, the predicted values obtained at the optimum US conditions (91% amplitude, 15 min) and the experimental values were compared. The experimental values obtained for TPC and anthocyanins were 1201.23 ± 13.06 mg GAE/100 g·dw and 379.12 ± 6.07 mg/100 g·dw, respectively. Antioxidant activity by ABTS was 6318.98 ± 76.84 µmol TE/100 g·dw and it was 9617.22 ± 120.92 µmol TE/100 g·dw for DPPH. According to the statistical analysis, no significant differences (*p* > 0.05) were observed between the predicted and experimental values. Therefore, the selected model was adequate to find the best US amplitude percentage and extraction time for processing blackberry residues.

### 2.5. Comparison of Extracts Obtained by Ultrasound versus Conventional Methodologies from Blackberry Residues

The extraction process of phenolic compounds can be significantly improved with cavitation using high power ultrasound by providing greater penetration of the solvent into the cellular material, and improving the transfer of compounds to and from the interfaces [30]. US extraction allows us to obtain a safe product without the use of solvents (methanol and ethanol, mainly) which may leave residues in the product, and generate environmental pollutants [31].

#### 2.5.1. Total Phenolic Content Extraction

**Figure 4:**
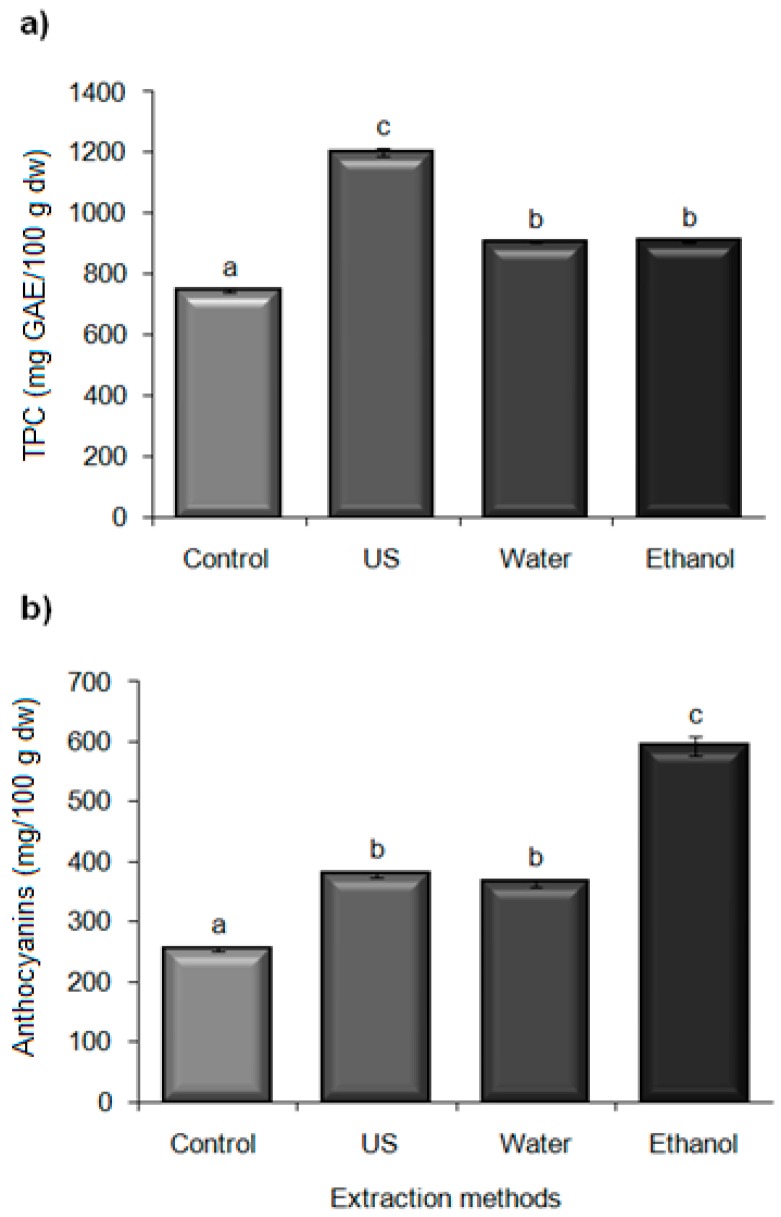
Extraction methods of (**a**) total phenolic content (mg gallic acid equivalent/100 g dry weight basis); and (**b**) anthocyanins (mg cyanidin-3-glucoside/100 g dry weight basis) from blackberry residues. US: ultrasonic extraction (91% amplitude by 15 min), Water: aqueous extraction, Ethanol: ethanolic extraction. ^a–c^ Different letters indicate a significant difference (*p* < 0.05) between methods.

During juice processing, a high proportion of soluble compounds are released, but in the residues the remaining antioxidant compounds are bonded to the dietetic fiber or proteins [32]. The US and the conventional extraction methods using water and an organic solvent (ethanol) were compared. Figure 4a shows the results for TPC extracted at the optimal US conditions (91% amplitude for 15 min) and by conventional methods (stirring the solid sample and solvent for 2 h). All extractions were significantly higher (*p* < 0.05) compared to the control sample (746.74 ± 5.69 mg GAE/100 g·dw). The US extraction yielded higher values (1201.23 ± 13.06 mg GAE/100 g·dw), in comparison with the use of solvents (water and ethanol; 904.49 ± 0.62 and 908.40 ± 3.01 mg GAE/100 g·dw, respectively) (Figure 4a), increasing the extraction of TPC from blackberry residues 32%.

Similar results were reported for apple residues in which ultrasound increased the TPC release (by 30%) in comparison to maceration [21]. In other applications, ultrasound has successfully increased swelling and hydration of a dry substrate, and thus the release of cell contents by enlarging the pores of the cell wall and increasing the cell disruption induced by ultrasound cavitation [33,34].

#### 2.5.2. Anthocyanins Extraction

Anthocyanins are water-soluble glycosides of anthocyanidins, normally found in the skin and responsible for the blue, red, purple and black colors of fruits. After juice extraction, many phenolic compounds, particularly anthocyanins, are still present in the solid residues and are suitable for extraction [35]. These compounds are polar molecules and the most common solvents used for their extraction are aqueous mixtures of methanol, ethanol or acetone [36]. Extraction efficiency is influenced by different factors, such as the solid-to-solvent ratio, the extraction time or the solvent composition [37,38]. Anthocyanins extracted by US and water were similar (*p* > 0.05) (379.12 ± 6.07 and 365.15 ± 7.29 mg/100 g·dw, respectively) and higher than the control (255.25 ± 3.50 mg/100 g·dw). Ethanol extraction was significantly more effective, reaching values of 593.58 ± 16.44 mg/100 g·dw (Figure 4b). These results agreed with those reported for black chokeberry wastes where ethanolic extraction of anthocyanins was more efficient than the ultrasonic-assisted process [35]. These results could be attributed to the polymerization of anthocyanins due to cavitation [39]. In addition, sonolysis of water may decrease anthocyanins content by inducing the formation of hydroxyl radicals (-OH) and hydrogen peroxide (H_2_O_2_) during cavitation, which also generates ring-opened products and chalcone aided by temperature increase during sonication [40,41].

#### 2.5.3. Antioxidant Activity of Extracts

**Figure 5:**
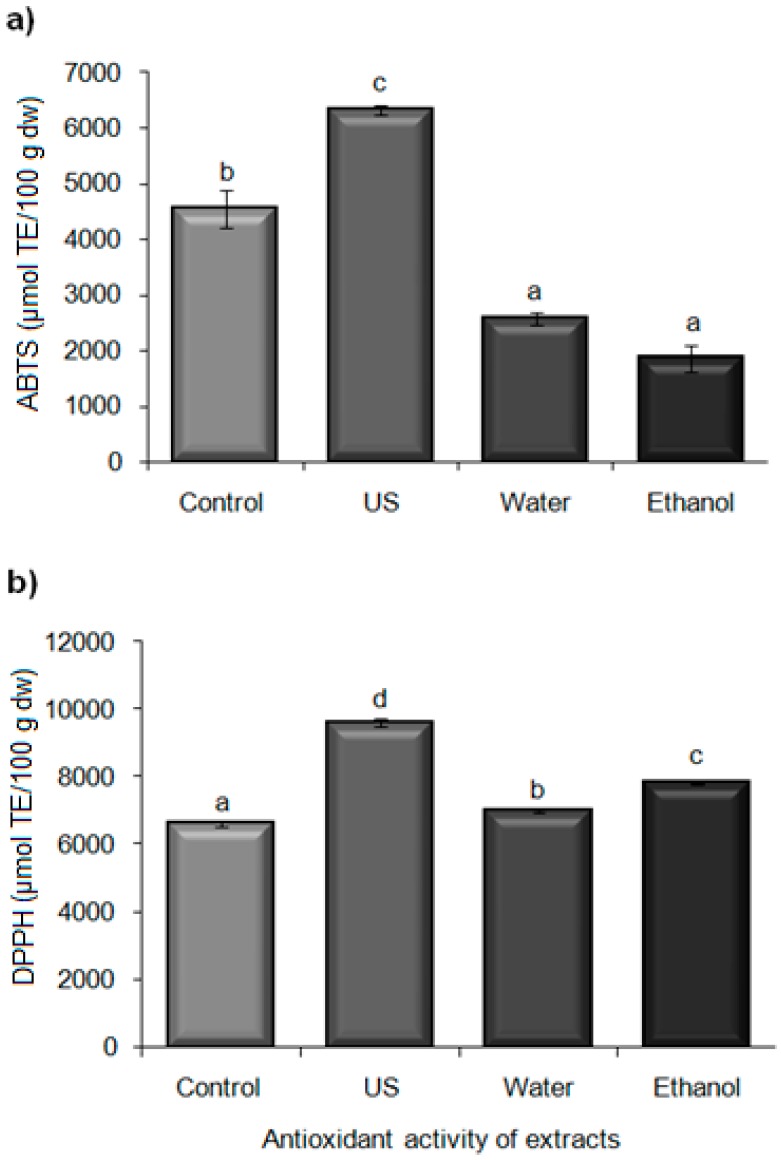
Antioxidant activity of extracts obtained by various methods: (**a**) ABTS (µmol Trolox equivalent/100 g dry weight basis) and (**b**) DPPH (µmol Trolox equivalent/100 g dry weight basis) from blackberry residues. US: ultrasound extraction (91% amplitude by 15 min), Water: aqueous extraction, Ethanol: ethanolic extraction. ^a–c^ Different letters indicate a significant difference (*p* < 0.05) between methods.

The results for ABTS and DPPH are shown in Figure 5. Water and ethanol extraction yielded similar antioxidant activity by ABTS (2567.91 ± 110.97 and 1857.65 ± 240.47 µmol TE/100 g·dw, respectively), and exhibited lower values compared with the control (4540.81 ± 333.27 µmol TE/100 g·dw), whereas the US extraction resulted in a higher antioxidant activity (6318.98 ± 76.84 µmol TE/100 g·dw) (Figure 5a). DPPH values exhibited a similar behavior to those of ABTS and were higher for the US treatment (9617.22 ± 120.92 µmol TE/100 g·dw) compared with the control, water and ethanol extraction (6623.03 ± 90.67; 6992.90 ± 39.77 and 7817.65 ± 15.48 μmoL TE/100 g·dw, respectively) (Figure 5b). The high antioxidant activity could be due to polyphenols which are polar compounds soluble in water that contain various OH groups trapping ABTS and DPPH free radicals. A good correlation between antioxidant activity by ABTS and phenolic compounds and anthocyanins (*R*^2^ = 0.824, *R*^2^ = 0.893, respectively) was observed, but this was not the case for DPPH, which suggests the presence of other antioxidants such as those found in berries’ seed oil [42]. On the other hand, factors such as the extraction time and presence of oxygen could also affect the content of these phenolic compounds [30]. In the present study, US extraction was carried out for 15 min whereas the other extraction methods required two hours.

## 3. Materials and Methods

### 3.1. Reagents

Folin–Ciocalteu 2N reagent (Sigma-Aldrich, St. Louis, MO, USA), anhydrous sodium carbonate (Meyer, Tláhuac, DF, Mexico), gallic acid (Meyer), potassium chloride (Sigma-Aldrich), anhydrous sodium acetate (Meyer), hydrochloric acid (Reasol, Iztapalapa, DF, Mexico), 2,2′azino-bis(3-ethylbenzthiazoline-6-sulphonic acid) diammonium salt (ABTS) ≥ 98% (Sigma-Aldrich), potassium persulfate crystals (Meyer), Trolox 97% (Sigma-Aldrich), absolute ethanol (Meyer), 1,1-di-phenyl-2-picrylhydrazyl (DPPH) (Sigma-Aldrich). All chemicals and reagents used in the study were of analytical grade (AG).

### 3.2. Instruments

Ultrasound (VCX-1500, Sonics & Materials, Inc., Newtown, CT, USA), industrial blender (38BL52 LBC10, Waring Commercial, Torrington, CT, USA), refrigerated centrifuge (Allegra 25™, Beckman Coulter, Palo Alto, CA, USA), lyophilizer (7753020, LABCONCO, Kansas City, MO, USA), mill analytical (IKA^®^ A11 basic, Wilmington, NC, USA), water bath (1210610, Cole-Parmer, Vernon Hills, IL, USA), overhead stirrer (HS-50A, Wisestir^®^ Wisd, laboratory instruments, Seongbuk-gu, Seoul, Korea), spectrophotometric microplate reader (Power Wave XS UV-Biotek, software Gen5 2.09, Winooski, VT, USA).

### 3.3. Sample Preparation

Blackberries (*Rubus fructicosus*) cultivars Tupy were obtained from a local market in Atotonilco el Grande, Hidalgo, México (geo-coordinates: 20°17′28″N, 98° 40′14″W, 2600 m above sea level). The plants were grown without any agronomical input and fruits are non-climacteric. Blackberries were harvested and processed in January 2015. Fruits without external injuries were selected and washed and processed in juice for obtaining residues. Juice was obtained by stirring the fruit at room temperature using an industrial blender and passing it through a conventional strainer to remove the bagasse (seeds and peel). Juice was then clarified by centrifugation at 15,300× *g* for 30 min, at 4 °C and further discarded, whereas the pulp (precipitate) was mixed with the bagasse previously separated. The residues (bagasse and pulp) were lyophilized, milled and sieved to obtain a particle size of 500 μm. The samples were stored in sealed plastic bags at −30 °C until further analysis.

### 3.4. Ultrasound Extraction

**Figure 6:**
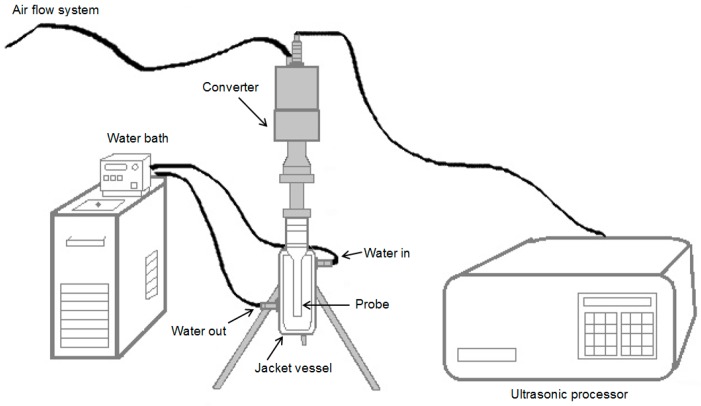
Ultrasonic equipment as a closed system used for ultrasonic extraction.

Extraction was performed using an ultrasound processor at 1500 W and a constant frequency of 20 kHz. A 4% solution (*w*/*v*) of lyophilized residues with deionized water was introduced in a jacket vessel through which water was circulated at 4 ± 1 °C in the secondary layer to reduce the heat generated during ultrasonic processing. The system was closed introducing a probe of 25 mm (amplitude transformer was connected between the converter and the probe), and an air flow system was connected to the converter to prevent its overheating (Figure 6). Amplitude levels of 80%–90% and time of 10–15 min with pulse durations of 2 s on and 4 s off were applied.

After treatment, samples reached temperatures of 25 ± 1 °C. Finally, treated samples were centrifuged at 15,300× *g* during 30 min at 4 °C. The extract obtained (supernatant) by ultrasound was placed in vials and frozen at −30 °C until analysis.

### 3.5. Experimental Design

Response surface methodology (RSM) is an effective statistical technique for optimizing complex processes. RSM reduces the number of experimental trials required to evaluate multiple parameters and their interactions [43]. In this study RSM was used to optimize and evaluate the effect of amplitude and time on the extraction of antioxidant compounds from blackberry residues. A central composite rotatable design (CCRD) for two independent variables at five levels was employed. The levels of the independent variables namely amplitude (X_1_, %) and ultrasonication time (X_2_, min) were selected based on preliminary experiments. Amplitude (X_1_) was varied between 80%–90%, and time (X_2_) between 10–15 min. The coded values for the independent variables were −α, −1, 0, +1, +α. The 13 experimental runs including five replicates at the center point were used. Experimental data were subjected to multiple nonlinear regression analysis using the JMP^®^ 5.1 statistical software (SAS Institute, Cary, NC, USA) to fit to a second-order polynomial model according to Equation (1):

(1) $$Yi=\beta_{0}+\sum_{i=1}^{2}\beta_{i}X_{i}+\sum_{i=1}^{2}\beta_{ii}X_{i}^{2}+\sum_{i}\sum_{j=i+1}\beta_{ij}X_{i}X_{j}$$

where *Y* is the predicted response, β_0_ the constant coefficient, β*_i_* the linear coefficient, β*_ii_* the quadratic coefficient, β*_ij_* is the cross product coefficients. *X_i_* and *X_j_* are independent variables. Three-dimensional response surface plots were obtained for each variable and optimized by superposing the contour plots generated using the SigmaPlot 10.0 graphing software (SYSTAT Software Inc., Richmond, CA, USA). The validity of the quadratic empirical model was tested by comparing the experimental and predicted values by *t*-test at confidence level of 95% using SPSS^®^ System for WIN™ version 15.0.

### 3.6. Comparison with Other Extraction Procedures

Optimized antioxidants extraction with ultrasound was compared with two solvent extraction methods using deionized water and ethanol. The lyophilized sample (20 g) was extracted 3 times with deionized water or ethanol using an overhead stirrer: 60 min (160 mL), 30 min (80 mL) and 30 min (80 mL) [44]. After each extraction the samples were centrifuged at 15,300× *g* during 30 min at 4 °C and supernatants were combined. A sample only mixed with deionized water, stirred for few seconds and centrifuged immediately was used as control. Supernatants were frozen at −30 °C until analysis.

### 3.7. Determination of Total Phenolic Content

Total phenolic content was determined by the Folin-Ciocalteau procedure [45]. Briefly, 100 µL of sample were mixed with 500 µL of 1:10 diluted Folin-Ciocalteu reagent. Then, 400 µL (7.5%) of sodium carbonate were added and the mixture incubated for 30 min at room temperature. The absorbance of the mixture was measured at 765 nm in the microplate reader. The linear standard curve was obtained with concentrations of 0, 100, 200 and 300 mg of gallic acid as a reference standard, and the results were expressed as mg of gallic acid equivalents per 100 g on a dry weight basis (mg GAE/100 g·dw).

### 3.8. Determination of Anthocyanins

The anthocyanins content was determined through the differential pH method [46]. Two buffer solutions were prepared: 0.025 M potassium chloride pH 1.0 and 0.4 M sodium acetate pH 4.5. The pH was adjusted by adding concentrated HCl. Blackberry extracts were diluted in these two buffer solutions. After 15 min of rest in the dark at room temperature, absorbance was measured at 510 and 700 nm using a microplate reader. The absorbance of the anthocyanins was calculated according to Equation (2):

(2) $$Abs=({Abs}_{510}-{Abs}_{700}){pH}_{1.0}-({Abs}_{510}-{Abs}_{700}){pH}_{4.5}$$

The concentration of anthocyanins in the extracts was calculated according to Equation (3):

(3) Anthocyanins(mg/L)=(Abs×MW×DF×1000)/ ε×0.52

where: *Abs*, absorbance; *MW*, molecular weight; *DF*, dilution factor; ε, molar absorptivity, considering the molar absorptivity (*ε*) of 26,900, the molecular weight of 449.2 g/moL for cyanidin-3-glucoside and the path length (0.52 cm) of the well. The results were expressed as mg of cyanidin-3-glucoside equivalent/100 g on a dry weight basis (mg cy-3-gl/100 g·dw).

### 3.9. Antioxidant Capacity

#### 3.9.1. ABTS Method

The radical cation (ABTS^•+^) was produced by reacting 7 mM ABTS stock solution with 2.45 mM potassium persulfate under dark conditions at room temperature for 16 h before use. The ABTS solution was diluted with deionized water to an absorbance of 0.70 ± 0.10 at 754 nm. After the addition of 20 µL of extract to 980 µL of diluted ABTS solution, absorbance readings were taken after incubation for 7 min at room temperature. The absorbance of the mixture was measured at 754 nm in a microplate reader. The standard curve was linear with concentration of 0, 50, 100, 200 and 300 µmol of Trolox. The antioxidant capacity was expressed as µmol Trolox equivalents per 100 g on a dry weight basis (µmol TE/100 g·dw) [47].

#### 3.9.2. Free Radical Scavenging Activity

Antiradical activity was measured with 1,1-diphenyl-2-picrylhydrazylradical (DPPH) [48]. An ethanolic solution (7.4 mg/100 mL) of the stable DPPH radical was prepared. Then, 100 µL of extract were taken into vials and 500 µL of DPPH solution were added, and the mixture was left to stand 1 h at room temperature. Finally, absorbance was measured at 520 nm using a microplate reader. The standard curve was linear with concentration of 0, 50, 100, 200 and 300 µmol of Trolox. Free radical scavenging activity was expressed as µmol of Trolox equivalents per 100 g on a dry weight basis (µmol TE/100 g·dw).

### 3.10. Statistical Analysis

All variables used to compare the optimized ultrasound extraction method with conventional methods were obtained by triplicate and expressed as mean ± standard deviation (SD). Data were analyzed performing a one-way analysis of variance (ANOVA) and differences among means were determined using a Tukey test with a level of significance of *p* < 0.05. For ultrasound values a Pearson correlations were used to determine the correlation between antioxidant activity and antioxidant compounds. The statistical package SPSS^®^ System for WIN™ version 15.0 was used.

## 4. Conclusions

The second-order polynomial model described the extraction of polyphenols and anthocyanins by ultrasound in blackberry residues. Optimization of the extraction conditions using a closed system allowed the obtention of maximum values for these compounds and antioxidant activity, and was successfully performed using response surface methodology. Our analysis demonstrated that time was the most influential factor that affected antioxidants extraction. US achieved a higher release of antioxidant compounds compared to the water and ethanol extraction methods. The results demonstrated that US is a valuable technology to extract phenolic compounds in a shorter time with greater yield without using toxic solvents. US is a promising technology for the recovery of added-value compounds from these residues, but further research is needed to evaluate the effect of ultrasound at a pilot-plant scale.

## Figures and Tables

**Table 1:** Combinations of amplitude and time with their coded terms obtained from RSM and their respective values of TPC, anthocyanins and antioxidant activity ^a^.

Run	Amplitude (%)	Time (min)	TPC ^b^ (mg GAE/100 g·dw)	Anthocyanins (mg/100 g·dw)	ABTS (μmol TE/100 g·dw)	DPPH (μmol TE/100 g·dw)
1	78 (−α)	12.5 (0)	1094.44 ± 70	345.53 ± 18	6953.86 ± 300	9237.42 ± 194
2	85 (0)	12.5 (0)	1201.24 ± 13	352.10 ± 29	6165.36 ± 645	8575.23 ± 485
3	90 (+1)	10 (−1)	1124.52 ± 31	340.25 ± 03	5601.75 ± 104	7815.00 ± 74
4	80 (−1)	10 (−1)	1311.19 ± 45	346.32 ± 02	5873.31 ± 166	9089.02 ± 22
5	85 (0)	9 (−α)	1438.14 ± 62	332.04 ± 07	3478.45 ± 604	7585.90 ± 108
6	85 (0)	16 (+α)	1433.79 ± 17	374.68 ± 01	4538.77 ± 499	7050.25 ± 163
7	85 (0)	12.5 (0)	1205.84 ± 84	356.66 ± 02	6124.96 ± 0	8557.20 ± 190
8	85 (0)	12.5 (0)	1220.31 ± 45	352.58 ± 01	6226.79 ± 270	8474.86 ± 417
9	92 (+α)	12.5 (0)	933.63 ± 39	357.52 ± 03	6841.88 ± 458	9219.67 ± 207
10	85 (0)	12.5 (0)	1245.28 ± 99	352.87 ± 00	6400.90 ± 834	8627.29 ± 542
11	80 (−1)	15 (+1)	1329.90 ± 18	358.91 ± 19	6185.99 ± 62	7593.98 ± 561
12	85 (0)	12.5 (0)	1226.87 ± 50	354.55 ± 03	6207.71 ± 333	8609.17 ± 340
13	90 (+1)	15 (+1)	1242.89 ± 101	378.32 ± 04	6234.75 ± 419	9571.14 ± 59

^a^ Values are the mean ± standard deviation (*n* = 3); ^b^ TPC: Total Phenolic Content.

**Table 2:** Regression coefficients of the predicted models for TPC, anthocyanins and antioxidant activity.

Term	Regression Coefficients			
	TPC	Anthocyanins	ABTS	DPPH
β_0_	1219.908 ^a^	353.752 ^a^	6225.144 ^a^	8568.75 ^a^
Linear				
β_1_	−52.030 ^c^	3.787 ^c^	−47.645	84.754
β_2_	16.366	13.870 ^a^	305.649 ^d^	−62.052
Quadratic				
β_11_	−84.905 ^a^	−0.236	466.540 ^c^	390.891 ^c^
β_22_	111.059 ^a^	0.680	−978.089 ^a^	−564.343 ^b^
Interaction				
β_12_	24.915	6.37 ^c^	80.08	812.795 ^a^
*R*^2^	0.97	0.97	0.94	0.95
Adj-*R*^2^	0.94	0.95	0.89	0.91
CV (%)	2.41	0.76	5.05	2.56

^a^ Significant at *p* < 0.0001; ^b^ Significant at *p* < 0.001; ^c^ Significant at *p* < 0.01; ^d^ Significant at *p* < 0.05.
